# Circadian disruption associated with nighttime light exposure contributes to airway epithelial remodeling via *TIMELESS* in childhood asthma

**DOI:** 10.3389/fped.2026.1788691

**Published:** 2026-04-23

**Authors:** Qiaoyan Dai, Fangxun Zhou, Tieshuai Liu, Hanghao Ma, Wei Zhou, Qidong Ye, Yingshuo Wang, Minfei Hu

**Affiliations:** 1 Department of Pediatrics, The First Affiliated Hospital of Ningbo University, Ningbo, China; 2 Department of Computer Science, Zhejiang University, Hangzhou, China; 3 Department of Anesthesiology, Sir Run Run Shaw Hospital, School of Medicine, Zhejiang University, Hangzhou, China; 4 Department of Intensive Care Unit, Ningde People’s Hospital, Ningde, China; 5 National Clinical Research Center for Child Health, The Children’s Hospital, Zhejiang University School of Medicine, Hangzhou, China

**Keywords:** artificial light at night (ALAN), childhood asthma, circadian rhythm, immune inflammation, TIMELESS gene

## Abstract

**Background:**

Childhood asthma is a common inflammatory airway disease, and its occurrence is influenced by environmental factors. Artificial light at night (ALAN) can disrupt circadian rhythms, but its relationship with childhood asthma remains unclear. To evaluate the association between ALAN and childhood asthma, and to investigate the role of the circadian clock gene in this relationship.

**Method:**

The study combined multiple approaches: an epidemiological analysis of asthma prevalence and urban ALAN levels in China; a pediatric asthma clinical study assessing nighttime light exposure, circadian disruption, and lung function; Mendelian randomization to test for a causal link between circadian disruption and asthma, and multi-omics plus experimental models to evaluate the function of ***TIMELESS***, a circadian clock gene.

**Results:**

ALAN exposure in Chinese cities has increased and was identified by AutoGluon modeling as a significant environmental risk factor for childhood asthma. In a pediatric asthma cohort, excessive nighttime light exposure was strongly associated with circadian rhythm disruption (*R* = 0.756, *p* < 0.001). Mendelian randomization analysis supported a causal relationship between circadian rhythm disruption and asthma (*p* = 0.040). Multi-omics analysis identified ***TIMELESS*** as a key circadian regulator that is highly expressed in the airway epithelium of asthmatic children. In an asthma mouse model, ***TIMELESS*** expression was upregulated in airway epithelium, and ***TIMELESS*** knockdown in airway epithelial cells significantly reduced their proliferation.

**Conclusions:**

Circadian disruption associated with nighttime light exposure may contribute to airway epithelial proliferation and remodeling via ***TIMELESS***, potentially exacerbating childhood asthma.

## Introduction

Asthma is a common chronic non-communicable disease marked by airway inflammation, bronchospasm, and airflow obstruction. It accounts for over 1% of global disability-adjusted life years (DALYs), with approximately 10% occurring in children and adolescents (1). In China, the incidence of childhood asthma is rising. Early-life wheezing is often triggered by airborne allergens and respiratory viruses, along with other environmental factors associated with asthma onset and severity (2). Continued exposure to these factors may impair lung development and contribute to persistent asthma into adulthood (3).

Among various environmental exposures, artificial light at night (ALAN) has emerged as a potential risk factor. ALAN refers to human-made lighting, both indoor and outdoor, that disrupts natural light-dark cycles. Nighttime light intensity above 10 lux is defined as light pollution (4). Currently, over 83% of the global population-including 99% in the United States and Europe-lives under light-polluted skies (5). Light pollution has expanded beyond cities into natural habitats, leading to a global rise in night sky brightness (6). This widespread ALAN significantly disturbs circadian rhythms in living organisms.

The circadian clock operates through interlocking transcription–translation feedback loops (TTFLs) that complete one cycle roughly every 24 h. Normal circadian rhythms are crucial for immune homeostasis. Immune cell activity and cytokine production follow daily cycles; when circadian timing is disrupted, immune regulation becomes impaired (7). In the lungs, many genes involved in inflammation and repair are under circadian control (8). Animal studies have shown that disruption of the biological clock can intensify airway inflammation and delay epithelial repair (9).

***TIMELESS*** (***TIM***) is a key component of the eukaryotic circadian regulatory system. Initially identified in Drosophila, ***TIM*** plays an essential role in the fly's circadian rhythm (10). Its mammalian homolog (m***TIM***) shares high sequence similarity with Drosophila ***TIMELESS2*** (***TIM***2) and is involved in DNA metabolism, chromosomal stability, and light-mediated circadian synchronization (11). In the mammalian brain, ***TIM*** exhibits rhythmic mRNA expression and physically interacts with core circadian proteins including ***mPER1/2/3*** and ***CRY1/2***, supporting its role as a functional clock protein (12).

Although previous research has suggested associations among environmental exposures, circadian biology, and inflammatory diseases, direct mechanistic evidence linking nighttime light pollution, circadian disruption, and asthma-associated immune responses is still limited. Therefore, we aimed to elucidate the intrinsic connections between nighttime light pollution, circadian rhythm disruption, and childhood asthma, with an emphasis on the circadian gene ***TIM***. Employing a multi-level methodology, we analyzed public data to evaluate childhood asthma prevalence relative to ALAN, assessed clinical data from asthmatic children, identified differential ***TIM*** expression in airway epithelial cells using single-cell RNA sequencing (scRNA-seq), and confirmed our findings through *in vivo* and *in vitro* experiments. Our research provides evidence that nighttime light exposure–related circadian disturbance may be associated with asthma-related molecular and cellular changes, thereby highlighting the potential relevance of circadian-based therapeutic interventions.

## Materials and methods

### Mice models

Six-week-old female SPF-grade C57BL/6J mice (16–18 g) were purchased from China Slack Laboratory Animal Co., Ltd. Mice were maintained under SPF conditions (20–26°C; 12-hour light/dark cycle) with free access to food and water. To induce asthma, mice received intranasal instillations of 50 μL HDM solution (10 mg/mL) on days 0, 7, and 14, while controls received saline (13). On day 17, all mice were euthanized by gradual-fill CO₂ inhalation in an uncharged chamber, with CO₂ delivered at a displacement rate of 30% of the chamber volume per minute. After loss of consciousness, death was confirmed, and cervical dislocation was performed as a secondary physical method prior to tissue collection. All procedures were approved by the Review Committee of Zhejiang University School of Medicine and in compliance with institutional guidelines (Ethics Code: ZJU20220416).

### HE and PAS staining

Lung tissues were fixed in 4% paraformaldehyde, dehydrated, embedded in paraffin, and sectioned (4 μm). HE staining was used to assess inflammatory infiltration and airway structure, while PAS staining evaluated goblet cell hyperplasia and mucus secretion. Images were obtained via light microscopy.

### Immunohistochemical (IHC) staining

Paraffin sections (4 μm) were deparaffinized and underwent antigen retrieval, followed by incubation with rabbit anti-TIM antibody (1:100, Proteintech) and HRP-conjugated goat anti-rabbit secondary antibody (Proteintech). Staining was visualized under light microscopy.

### Gene knockdown

Adenoviral vectors targeting ***TIM*** (sh-***TIM***) and negative control vectors (sh-NC) were designed by He Yuan Biologicals. BEAS-2B cells were infected with either sh-***TIMELESS*** or sh-NC adenoviruses (MOI = 50) and selected with 5 μg/mL puromycin to obtain stable knockdown cell lines for subsequent proliferation assays.

### Edu cell proliferation

BEAS-2B cells (5 × 10^3^/well) were seeded in 96-well plates and treated with 10 μg/mL HDM or left untreated. The next day, cells were incubated with 50 μM EdU medium, fixed, neutralized (2 mg/mL glycine), and permeabilized (0.5% Triton X-100). EdU was detected using 1× Apollo solution, and nuclei were stained with Hoechst 33,342. Five random fields were imaged by fluorescence microscopy, and proliferation was calculated as:Cell proliferation rate (%) = (Number of EdU-positive cells/Total number of nuclei) × 100.

### Patient selection and data collection

This retrospective study included children with asthma who attended follow-up visits at the pediatric outpatient clinic of the First Affiliated Hospital of Ningbo University between January 2022 and June 2024. Inclusion criteria were: age <16 years, diagnosis meeting standard clinical criteria for asthma, and a follow-up period ≥6 months. Patients with incomplete data, including missing pulmonary function tests, laboratory results, or questionnaires, were excluded. Lung function was assessed by spirometry, and children with forced expiratory volume in one second (FEV₁) < 80% of the predicted value were classified into the impaired group.

Parents of eligible participants completed structured questionnaires assessing residential floor level, smartphone usage (Smartphone Addiction Proneness Scale), nighttime light exposure, and sleep quality (Pittsburgh Sleep Quality Index, PSQI). The study was approved by the Ethics Review Board of the First Affiliated Hospital of Ningbo University (Approval No. 2024-IRB-245RS). Informed consent was waived in accordance with the Declaration of Helsinki, as all data were anonymized and posed minimal risk.

### Global burden of disease (GBD) data analysis

Asthma incidence data for children under 14 from 2000 to 2020 were extracted using the GBD Results Tool (http://ghdx.healthdata.org/gbd-results-tool) (14). Age-standardized rates (ASRs) were used as the primary metric. Temporal trends in ASR were assessed using the Estimated Annual Percentage Change (EAPC). A regression line was fitted to the natural logarithm of the rates, expressed as *y*  = *α* + *β*x + ɛ, where *y* = ln(ASR) and *x* = calendar year. The EAPC was calculated as100 × [exp(*β*)−1], with its 95% CI derived from the linear regression model. Population-wide Summary Exposure Values (SEVs) and demographic data for the same period in China were also extracted, including 68 variables representing environmental and health risk factors.

### Public data collection

Environmental and socioeconomic data were obtained from open-access sources. Nighttime light data (2000–2020) were sourced from the PANDA-China dataset, based on DMSP-OLS satellite images processed with a Convolutional Long Short-Term Memory Network (CLSMN) algorithm (https://doi.org/10.11888/Socioeco.tpdc.27120) (15). Regional GDP data were obtained from the National Bureau of Statistics (https://www.stats.gov.cn/sj/). PM2.5 levels were derived from the high-resolution near-surface air pollutant dataset of the National Tibetan Plateau Science Data Center. Industrial pollution data—including sulfur dioxide, wastewater, and dust emissions—were extracted from the China Urban Statistical Yearbook.

### Autogluon model construction

Machine learning modeling was conducted using the AutoGluon framework. The AutoGluon.Tabular package (v1.1.1, Python 3.11.8) was used for training and evaluation (https://auto.gluon.ai/dev/index.html) (16). The framework incorporated models such as CatBoost, LightGBM, XGBoost, RandomForestMSE, ExtraTreesMSE, NeuralNetFastAI, and ensemble methods. Variable importance scores were used to assess each feature's contribution (https://explained.ai/rf-importance/).

### Mendelian randomization analysis (MR)

MR analysis was conducted to explore the causal relationship between circadian rhythm traits and pediatric asthma. Chronotype trait data were obtained from a GWAS meta-analysis conducted by Neale Lab (GWAS ID: ukb-a-11), involving 301,143 European individuals and 10,894,596 SNPs. Pediatric asthma outcome data were obtained from a separate GWAS dataset (GWAS ID: finn-b-ASTHMA_CHILD), which included 3,025 children with asthma and 135,449 controls, with a total of 16,379,865 SNPs. To minimize linkage disequilibrium (LD) bias, SNPs were clustered using a threshold of r^2^ = 0.001 within a 10,000 kb window. A total of 87 instrumental variables (IVs) meeting the significance threshold (*p* < 5 × 10⁻⁸) were identified for MR analysis. The Inverse Variance Weighted (IVW) method was employed to assess causal effects under the assumption of no directional pleiotropy (17). Two-sample MR analyses were performed using the TwoSampleMR (v0.5.6) and MR-PRESSO (v1.0.0) packages in R.

### Public dataset collection

Sequencing data were retrieved from the Gene Expression Omnibus (GEO) database (https://www.ncbi.nlm.nih.gov/geo/), including two microarray datasets (GSE40732, GSE40888) and a scRNA-seq dataset (GSE193816). The GSE40732 dataset contained PBMCs from 97 atopic asthmatic and 97 non-atopic, non-asthmatic children. The GSE40888 dataset included PBMC samples from 14 allergic asthmatic, 8 non-allergic asthmatic, and 14 healthy children, treated with anti-CD3/CD28, LpA-stimulated, or left unstimulated. The GSE193816 dataset included lung cells collected via endobronchial brushing from allergic asthmatics (*n* = 4) and allergic controls (*n* = 4), analyzed using scRNA-seq for cell-type-specific expression profiling.

### Differential expression analysis

In GSE40732, Differentially expressed genes (DEGs) were identified using the limma package with thresholds of |logFC| > 0.25 and *p* < 0.05. In GSE40888, weighted gene co-expression network analysis (WGCNA) was used. After filtering with “goodSamplesGenes”, a soft-threshold *β* = 9 was applied, yielding 11 gene modules. Modules associated with disease or stimulation were identified via eigengene analysis.

For GSE193816, scRNA-seq data were processed using Seurat (v5.1.1) with SCT normalization and canonical correlation analysis. The top 3,000 variable genes and 20 principal components were used for clustering with “FindClusters”, and resolution was optimized via clustree. Marker genes were identified with “FindAllMarkers”. Cell-cell communication was assessed using CellChat and a ligand-receptor database. Circadian rhythm gene sets included 13 clock-controlled and 35 core clock genes (18, 19).

### Statistical analysis

All analyses were conducted in R (v4.1.2). Normally distributed data were expressed as mean ± SD and compared using *t*-tests; non-normal data were summarized by the interquartile range (IQR) and compared using the Mann–Whitney *U*-test. Categorical variables were analyzed via chi-square or Fisher's exact tests. Correlations were evaluated with Pearson coefficients. A two-sided *p* < 0.05 was considered statistically significant. Statistical significance was determined based on *p*-values, with the following thresholds applied: *p < 0.05, **p < 0.01, and ***p < 0.001.

## Results

### Association between nighttime light pollution and the prevalence of childhood asthma in China

**Figure 1:**
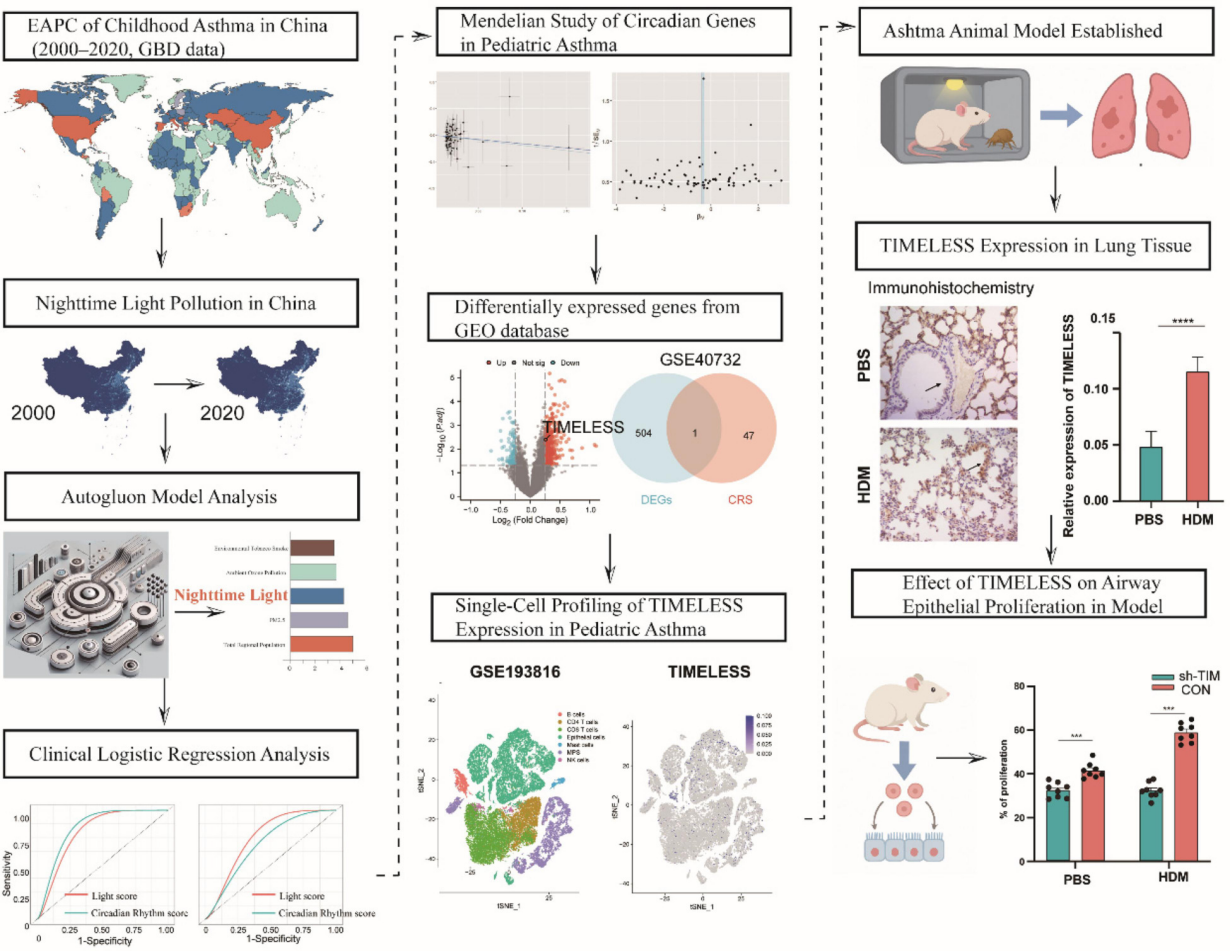
Workflow of the study. This study comprises four parts:Modeling the association between nighttime light pollution and pediatric asthma;Assessing the impact of light pollution on circadian rhythms;Bioinformatic analysis linking circadian genes to pediatric asthma;Validation of ***TIMELESS*** function in airway epithelial proliferation in an HDM-induced asthma model.

**Figure 2:**
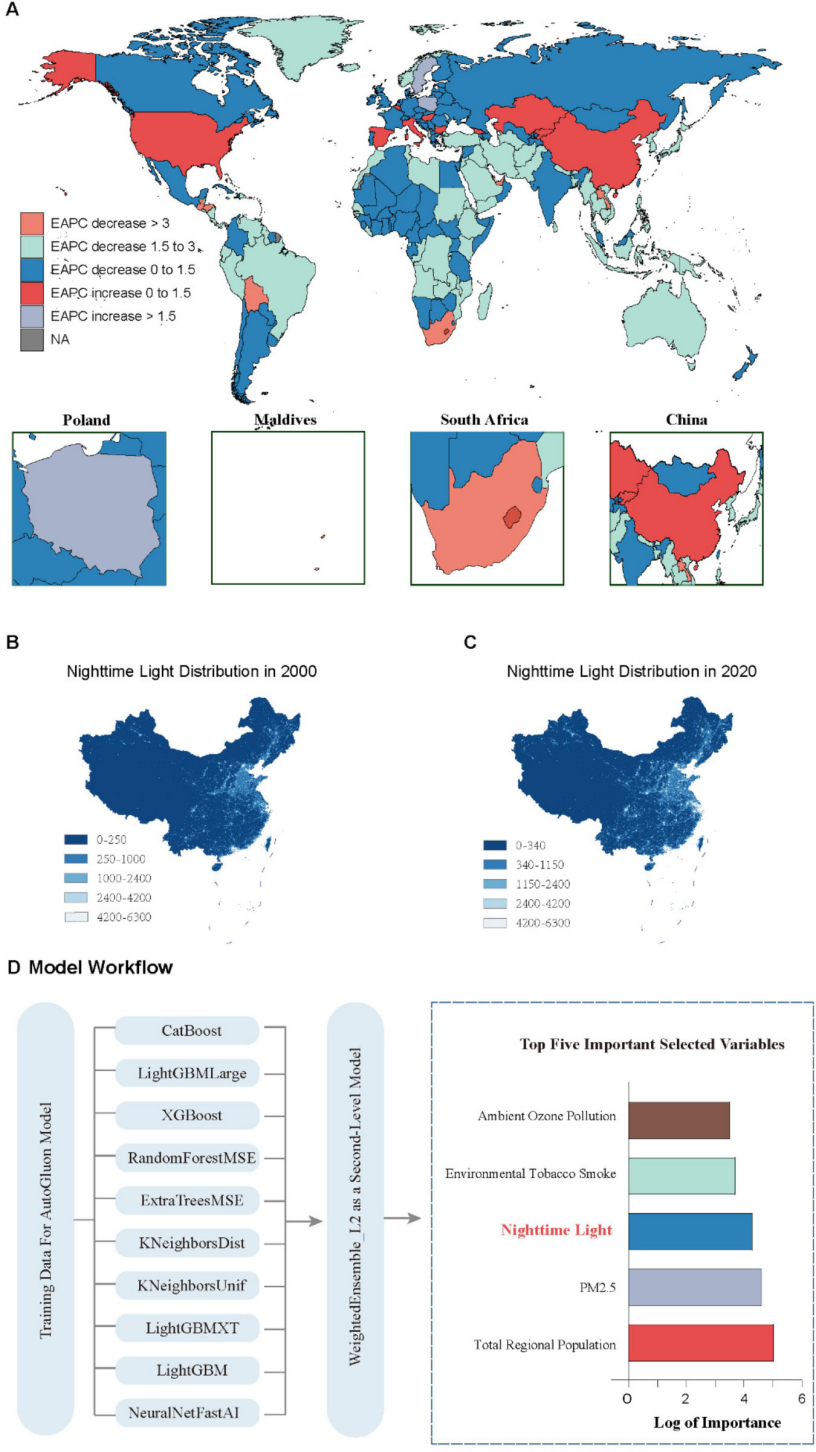
Nighttime light pollution and childhood asthma incidence. **(A)** Estimated Annual Percentage Change (EAPC) in the incidence of childhood asthma worldwide from 2000 to 2020 based on the GBD database. **(B)** Distribution of nighttime lighting across China in 2000 from the PANDA-China database. **(C)** Distribution of nighttime lighting across China in 2020 from the PANDA-China database. **(D)** AutoGluon model analysis identifying risk factors for childhood asthma in China. Variables are ranked by Importance, with nighttime light pollution ranked third.

The flowchart of our study was shown in Figure 1. Using GBD data, we observed a rising global trend in childhood asthma. In China, the age-standardized prevalence showed a consistent increase from 2000 to 2020, with an EAPC of 0.321% (95% CI: −0.354 to 1.001%) (Figure 2A). Concurrently, analysis of PANDA-China nighttime light data revealed a substantial increase in light pollution, with average intensity rising from 89.42 nW/cm^2^/sr in 2000 (Figure 2B) to 211.22 nW/cm^2^/sr in 2020 (Figure 2C).

To evaluate environmental risk factors, we integrated asthma ASR data with variables including SEV, nighttime lighting, GDP, population, PM2.5, and industrial pollution. AutoGluon modeling identified the WeightedEnsemble_L2 as the optimal Level 2 model (Figure 2D). Variable importance analysis ranked nighttime light pollution as the third strongest predictor of childhood asthma—after population and PM2.5—with an importance score of 19,670.02 (*p* = 0.018), highlighting its relevance as a significant environmental risk factor.

### Effects of nighttime light pollution on children's circadian rhythms

**Figure 3:**
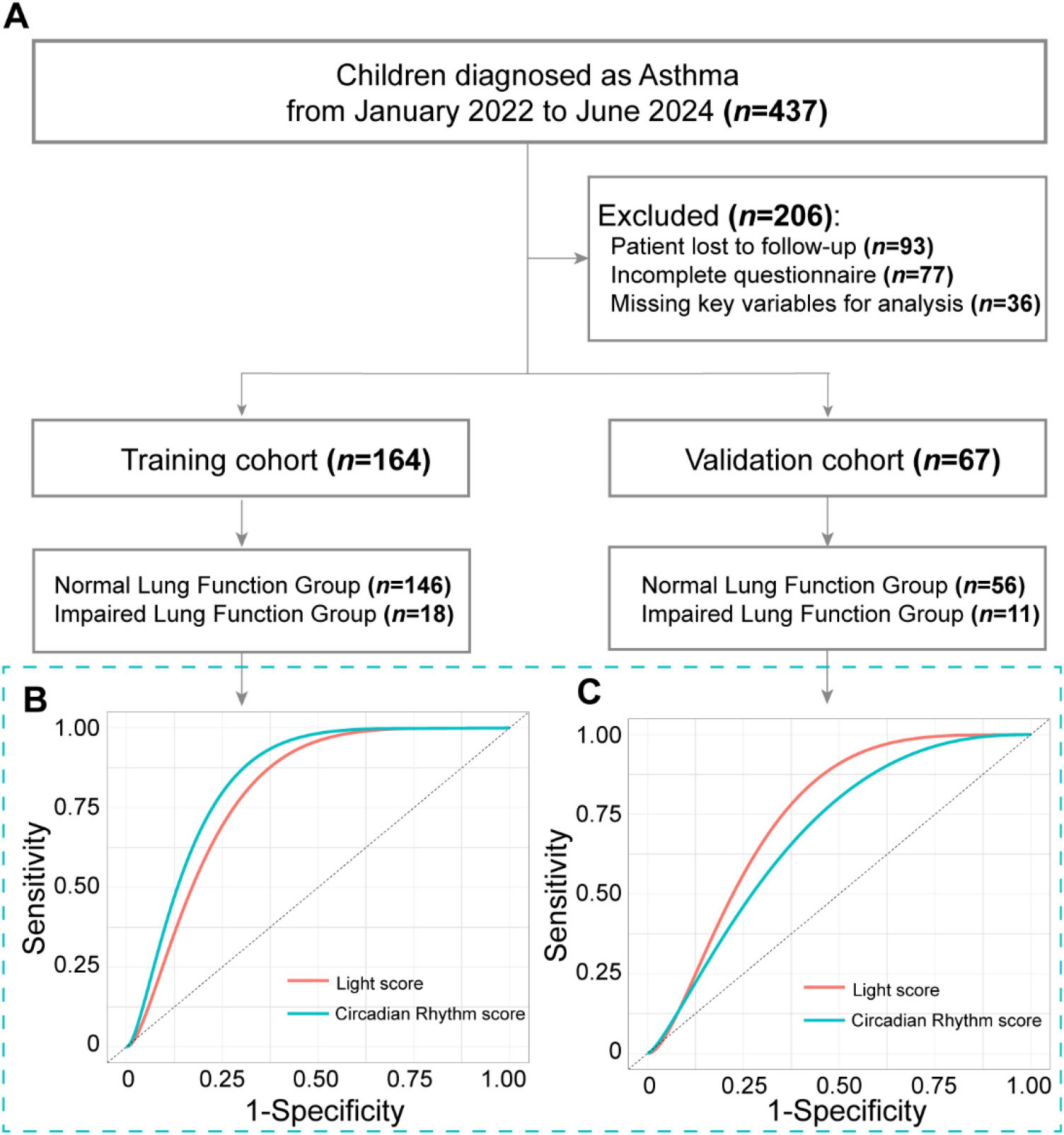
Clinical analysis of children with asthma. **(A)** Flowchart depicting the screening and grouping process for children with asthma. **(B)** Receiver Operating Characteristic (ROC) curves illustrating the predictive performance of the Circadian Rhythm Score and Light Score in the training cohort. **(C)** ROC curves illustrating the predictive performance of the Circadian Rhythm Score and Light Score in the validation cohort.

A retrospective analysis of 437 pediatric asthma follow-up cases identified 231 children with confirmed asthma. Participants were randomly assigned to a training set (*n* = 164) and a validation set (*n* = 67) in a 7:3 ratio. Based on pulmonary function test results, children were categorized into the Impaired Group or the Normal Group (Figure 3A).

Sleep quality, reflecting circadian rhythm status, was assessed using the PSQI completed by parents and used as the Circadian Rhythm Score. Nighttime light exposure was quantified via a Light Score, derived from a linear model combining the Smartphone Addiction Proneness Scale and the Nighttime Light Use Questionnaire. Higher scores indicated poorer sleep quality and greater light exposure, respectively.

Univariate analysis showed both scores were significantly higher in the Impaired Group across training and validation sets (Table 1). Logistic regression confirmed both as significant risk factors for impaired lung function (Light Score: OR = 1.219, 95% CI: 1.014–1.465, *p* = 0.035; Circadian Rhythm Score: OR = 1.214, 95% CI: 1.018–1.449, *p* = 0.032).

**Table 1:** Characteristics of the enrolled asthma patients.

Characteristics	Training cohort *n* = 164, No. (%)			Validation cohort *n* = 67, No. (%)		
	Normal Group (*n* = 146)	Impaired Group (*n* = 18)	*p*-value	Normal Group (*n* = 56)	Impaired Group (*n* = 11)	*p*-value
Age, Mean (SD), year	8.79 (2.59)	9.67 (1.85)	0.082	9.09 (2.95)	10.36 (2.11)	0.105
Weight, Mean (SD), kg	32.22 (12.14)	37.89 (7.27)	0.055	31.35 (10.62)	37.64 (6.85)	0.064
Gender (male)	96.00 (65.75)	16.00 (88.89)	0.085	26.00 (46.43)	7.00 (63.64)	0.475
White Blood Cell, Mean (SD), *10^9^/L	7.14 (1.96)	8.64 (1.88)	0.002*	6.92 (2.15)	8.26 (1.48)	0.052
Neutrophil, Median (IQR), *10^9^/L	3.52 (2.46–3.99)	5.68 (2.93–5.68)	0.036*	3.00 (2.46–3.98)	5.69 (4.30–6.03)	0.007*
Hemoglobin, Mean (SD), g/L	134.63 (9.04)	127.00 (7.99)	<0.001***	134.25 (8.76)	127.82 (9.04)	0.030*
Platelets, Mean (SD), *10^9^/L	304.79 (78.28)	313.39 (35.85)	0.424	290.04 (82.09)	310.91 (45.94)	0.249
C-reaction Protein, Median (IQR), mg/L	0.79 (0.10–3.00)	3.27 (1.85–7.00)	0.002*	0.74 (0.10–3.00)	3.55 (1.85–5.50)	0.001*
Eosinophil, Median (IQR), *10^9^/L	0.35 (0.21–0.61)	0.65 (0.44–1.12)	0.004*	0.38 (0.23–0.60)	0.65 (0.32–0.66)	0.172
FEV1/FVC, Mean (SD), %	98.44 (11.68)	75.91 (3.37)	<0.001***	100.51 (12.20)	71.52 (6.75)	<0.001***
PEF, Mean (SD), L/min	75.71 (20.33)	74.83 (21.89)	0.865	76.81 (19.44)	72.08 (16.79)	0.455
FEF25, Mean (SD), L/s	75.12 (20.03)	63.52 (27.15)	0.027*	74.05 (19.91)	57.97 (18.12)	0.016*
FEF50, Median (IQR), L/s	67.90 (50.30–81.80)	43.10 (27.10–57.20)	<0.001***	70.87 (23.48)	45.66 (19.56)	0.001*
FEF75, Median (IQR), L/s	60.90 (38.55–83.00)	32.20 (12.10–46.67)	<0.001***	67.44 (29.78)	33.51 (16.23)	<0.001***
FeNo, Median (IQR), ppb	20.50 (15.00–44.00)	43.00 (31.00–59.00)	0.005*	25.50 (16.50–44.00)	31.00 (17.00–43.00)	0.741
IgE, Median (IQR), IU/mL	304.00 (189.00–586.00)	313.00 (199.00–364.00)	0.476	328.50 (113.75–586.00)	586.00 (281.50–657.00)	0.167
Residential floor height, Mean (SD)	107.00 (73.29)	7.00 (38.89)	0.007**	39.00 (69.64)	5.00 (45.45)	0.231
Light Score, Mean (SD), Median (IQR)	7.00 (5.00–12.25)	15.00 (12.00–18.75)	<0.001***	7.00 (5.00–15.00)	15.00 (12.00–18.00)	0.008*
Circadian Rhythm Score, Median (IQR)	5.50 (3.00–10.50)	13.00(9.00–16.00)	<0.001***	8.00(3.00–13.00)	11.00(10.50–14.50)	0.043*

Data are presented as Mean (standard deviation, SD) or Median (interquartile range, IQR), as appropriate.White blood cell, neutrophil, eosinophil, and platelet counts are expressed in *10⁹/L; hemoglobin in g/L; C-reactive protein in mg/L; FeNO in parts per billion (ppb); and IgE in IU/mL.

FEV1/FVC, forced expiratory volume in one second/forced vital capacity; PEF, peak expiratory flow; FEF25/50/75, forced expiratory flow at 25%, 50%, and 75% of the pulmonary volume; FeNO, fractional exhaled nitric oxide; Light Score: composite score derived from the Smartphone Addiction Proneness Scale and Nighttime Light Use Questionnaire; Circadian Rhythm Score: calculated using the Pittsburgh Sleep Quality Index (PSQI); Residential floor height is recorded as average floor level (stories). * *p* < 0.05, ** *p* < 0.01, ***p < 0.001.

Receiver operating characteristic (ROC) curve analysis demonstrated superior predictive performance for the Light Score. In the training set, the AUCs were 0.798 for the Light Score and 0.746 for the Circadian Rhythm Score (Figure 3B). In the validation set, AUCs were 0.837 and 0.689, respectively (Figure 3C). Spearman correlation revealed a strong positive association between the two scores (R = 0.756, *p* < 0.001), supporting a link between nighttime light exposure, circadian disruption, and lung function impairment in asthmatic children.

### Circadian rhythm genes and childhood asthma

**Figure 4:**
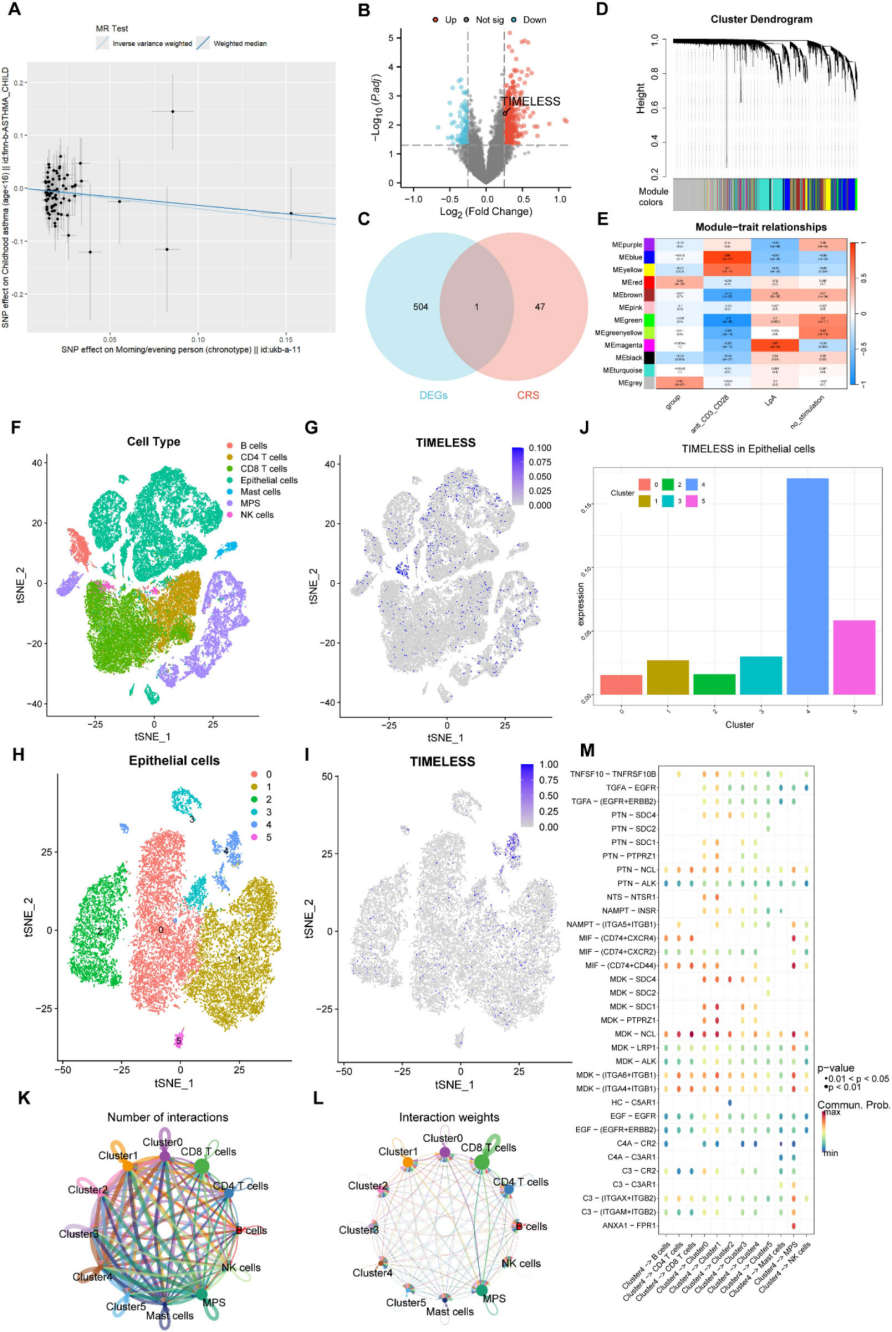
***TIMELESS*** expression and Its potential mechanism in childhood asthma. **(A)** Mendelian Randomization (MR) analysis demonstrating the relationship between circadian rhythm disturbances and childhood asthma. **(B)** Volcano plot showing differentially expressed genes in the GSE40732 dataset. **(C)** Venn diagram illustrating the intersection of differentially expressed genes from the GSE40732 dataset with genes associated with circadian rhythm. **(D)** Weighted Gene Co-expression Network Analysis (WGCNA) results from the GSE40888 dataset. **(E)** Distribution of 11 gene modules identified via WGCNA in the GSE40888 dataset. **(F)** Unbiased clustering analysis of cells in the GSE193816 dataset using t-distributed Stochastic Neighbor Embedding (tSNE) visualization. **(G)** Expression profile of the ***TIMELESS*** gene across distinct cell populations in the GSE193816 dataset. **(H)** Unbiased clustering analysis of epithelial cells in the GSE193816 dataset, revealing six distinct clusters visualized via tSNE mapping. **(I)** tSNE visualization of ***TIMELESS e***xpression across different epithelial cell clusters. **(J)** Bar plot comparing ***TIMELESS*** expression levels across epithelial cell clusters. **(K)** Cell communication analysis illustrating interaction frequency between Cluster 4 and other clusters. **(L)** Weighted cell communication analysis evaluating the interaction strength between Cluster 4 and other clusters. **(M)** Cell-Ligand-Receptor (CLR) network analysis depicting the interaction landscape between Cluster 4 and other clusters.

To evaluate the causal link between circadian rhythm disruption and childhood asthma, MR analysis was performed. The Cochran *Q* test showed no significant heterogeneity (*Q* = 66.40, *p* = 0.843), confirming the consistency of instrumental variables. IVW analysis revealed a significant causal association (OR = 0.678, 95% CI: 0.468–0.983, *p* = 0.040) (Figure 4A). MR-Egger regression showed no directional pleiotropy (intercept = −0.014, SE = 0.009, *p* = 0.131), supporting the robustness of the findings. These results suggest that circadian rhythm disruption may contribute to asthma development, underscoring the importance of limiting nighttime light exposure.

In the GSE40732 dataset, 505 DEGs were identified in asthmatic children (393 upregulated, 112 downregulated) (Figure 4B). Cross-referencing with CRS identified ***TIM*** as the only overlapping gene (Figure 4C). WGCNA of GSE40888 identified 11 gene modules (Figures 4D,E), with the yellow module significantly associated with inflammation and immunity. Among six hub genes in this module (***TIM, AUTS2***, ***NPFFR2***, ***TIPIN, REV1***, ***SIRT1***), ***TIM*** was most strongly linked to both circadian regulation and asthma.

Single-cell RNA-seq analysis (GSE193816) showed elevated ***TIM*** expression in airway epithelial cells (Figures 4F,G), particularly in Cluster 4, as revealed by tSNE analysis (Figures 4I,J). Cell communication analysis indicated Cluster 4 had increased interaction frequency and strength with other epithelial subsets (Figures 4K,L).

To elucidate ***TIM***-related signaling, Cell-Ligand-Receptor network analysis identified key ligand-receptor pathways involving Cluster 4, including TGFA–EGFR, EGF–EGFR, NAMPT–INSR, PTN–NCL, and MDK–NCL—pathways linked to cell proliferation, growth, and repair. These findings suggest ***TIM*** may promote epithelial remodeling and inflammation in childhood asthma.

### TIM in asthma and Its effects on cell proliferation

**Figure 5:**
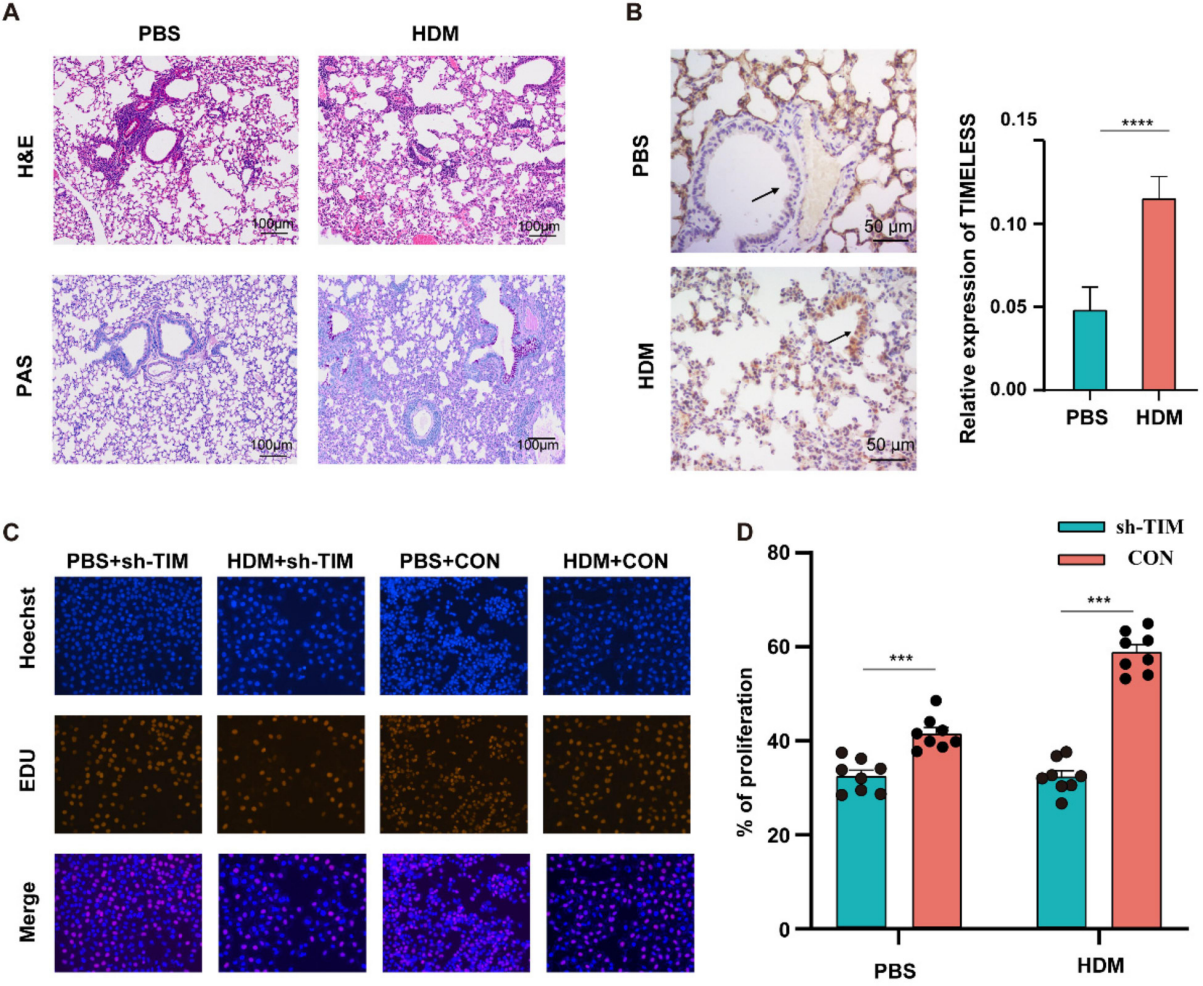
Experimental validation of ***TIMELESS*** in asthma. **(A)** Representative images of Hematoxylin and Eosin (H&E) staining and Periodic Acid-Schiff (PAS) staining in lung tissues from PBS and HDM-treated mice. **(B)** Immunohistochemistry (IHC) staining visualizing ***TIMELESS*** protein expression in lung tissues from PBS-treated (control) and HDM-treated (asthma model) mice. **(C)** Comparison of epithelial cell proliferation in PBS, HDM, and ***TIMELESS***-knockdown groups. **(D)** Bar plot summarizing cell proliferation levels across experimental groups as assessed by the EdU assay.

To validate the asthma model, histological analysis was first performed. H&E staining revealed typical airway remodeling in HDM-challenged lungs, including wall thickening, inflammatory infiltration, and alveolar narrowing. PAS staining confirmed goblet cell hyperplasia and increased mucus secretion compared to PBS controls (Figure 5A), indicating successful model induction.IHC localized ***TIM*** expression mainly to the airway epithelium, with markedly higher levels in the HDM group (Figure 5B).

To explore ***TIM***'s functional role, a stable ***TIM*** knockdown cell line (sh-***TIM***) was generated in mouse airway epithelial cells. EdU assays showed significantly reduced proliferation in both PBS- and HDM-treated sh-***TIM*** cells (*p* < 0.001; Figures 5C,D).

Collectively, these findings indicate that ***TIM*** may be involved in airway epithelial cell proliferation and may potentially contribute to airway remodeling and mucus hypersecretion, which are processes associated with asthma pathogenesis.

## Discussion

This study provides the first comprehensive evidence linking ALAN to childhood asthma, from broad epidemiological trends down to cellular mechanisms. By integrating population data with cohort findings and experimental models, we demonstrated that increasing nighttime light pollution is associated with a higher risk of asthma in children, likely through circadian rhythm disruption. We found that urban areas in China with greater ALAN intensity have elevated childhood asthma prevalence, identifying light pollution as a significant environmental risk factor. At the individual level, children in a high-ALAN exposure cohort showed pronounced circadian disruption alongside worse lung function, and a Mendelian randomization analysis supported a causal link between circadian misalignment and asthma development. Moreover, our molecular analyses demonstrated that the circadian clock gene ***TIM*** was upregulated in the airway epithelium of asthmatic mouse models, and this upregulation may be associated with airway epithelial remodeling. Collectively, our multi-level evidence suggests that nighttime light pollution may promote airway remodeling in pediatric asthma through circadian rhythm disruption.

Severe asthma is relatively uncommon among children (20). Approximately 60% of individuals diagnosed with asthma before the age of 10 experience remission, whereas remission rates in adult-onset asthma range from only 5% to 15% (21, 22). However, repeated environmental exposure during childhood may impair airway development and reduce maximal lung function, potentially resulting in persistent asthma symptoms into adulthood (23, 24).With the rapid growth of urbanization and lighting technology, China's nighttime artificial illumination intensity and coverage have increased dramatically, with reported annual growth rates of around 6.5% (25). While night lighting improves convenience and extends productive hours, it also introduces a new form of environmental pollution (chronic light exposure, also known as ALAN), which has become one of the fastest growing environmental problems globally (26). An increasing body of research suggests that chronic exposure to ALAN can directly disrupt circadian rhythms and sleep patterns, suppress nocturnal melatonin, and perturb peripheral clock gene expression, thereby modulating immune function and airway inflammation. Such circadian disruption may specifically increase susceptibility to allergic diseases including asthma, highlighting light pollution as a potential environmental contributor to pediatric asthma risk (27).

In our AutoGluon analysis, nighttime light pollution emerged as a significant predictor of childhood asthma prevalence, ranking just behind established risk factors such as population density and ambient PM2.5 pollution (2, 28).This finding aligns with previous reports linking ALAN exposure to increased allergic disease prevalence, particularly adolescent asthma and allergic rhinitis. The proposed mechanism involves ALAN-induced suppression of melatonin secretion and disruption of circadian gene expression, impairing immune regulation and increasing inflammation susceptibility (27) In our pediatric cohort, high nocturnal light exposure correlated with notable circadian rhythm disturbances. Mendelian randomization analysis supported a causal role of circadian disruption in asthma development, consistent with occupational studies demonstrating increased asthma risk among night-shift workers whose schedules misalign with their circadian preferences (29).These observations suggest that misalignment between external day-night cycles and an individual's internal circadian clock can facilitate the development of childhood asthma.

The mammalian circadian system comprises a master clock in the hypothalamic suprachiasmatic nucleus (SCN) and peripheral clocks throughout the body.Key circadian genes driving these rhythms include ***CLOCK, BMAL1, PER3***, and ***TIM***.Notably, genetic polymorphisms in several circadian-related genes have been associated with asthma susceptibility. For example, variants in core clock genes such as ***TIM*** have been found at higher frequencies in children with asthma compared to healthy individuals. This genetic link further underscores the relevance of circadian clock dysfunction in asthma. Circadian rhythms regulate pulmonary immune responses and airway epithelial functions, maintaining daily fluctuations of inflammatory mediators (30). Animal studies demonstrate that disrupted circadian rhythms exacerbate airway inflammation in asthma models (31). Additionally, core circadian genes like ***PER2*** regulate immune responses; ***PER2***-deficient mice exhibit heightened inflammatory reactions due to dysregulated immune activity (31). Thus, disruption of circadian gene expression can compromise immune homeostasis, potentially intensifying airway inflammation.

Our research group has previously characterized airway remodeling in our model using single-cell transcriptomics (32). In this study, our multi-omics analysis identified ***TIM*** as a critical mediator linking circadian disruption to asthma exacerbation. ***TIM*** was originally identified in Drosophila as a core circadian clock component, and in mammals it has also been shown to participate in regulating the molecular clock complex (33). Our study revealed elevated ***TIM*** expression in airway epithelial cells of asthmatic children, indicating its role in airway responses to circadian disturbances. Similarly, Guo et al. reported significantly increased ***TIM*** expression in an asthma mouse model, highlighting airway circadian reprogramming in response to chronic inflammation (34).

This reprogramming of the molecular clock in the airway may be a reaction to chronic inflammatory stimulation. During the repeated injury–repair cycles of the airway epithelium in chronic asthma, the expression of clock genes like ***TIM*** could be activated as the body attempts to coordinate epithelial cell proliferation and tissue repair processes. However, such an adjustment might prove to be a double-edged sword. On one hand, upregulation of ***TIM*** might enhance the survival of damaged epithelial cells and facilitate DNA repair; on the other hand, excessive ***TIM*** could disrupt the normal circadian control of the cell cycle, leading to aberrant epithelial proliferation and airway remodeling. In colorectal cancer models, for example, ***TIM*** is often overexpressed to support the rapid proliferation of tumor cells, and knocking out ***TIM*** results in the accumulation of DNA damage and G₂/M cell-cycle arrest, thereby inhibiting cell proliferation (35). Consistent with these findings, our mouse model showed that ***TIM*** silencing significantly reduced airway epithelial cell proliferation, suggesting potential therapeutic targets for mitigating airway remodeling in childhood asthma.

This study has several limitations. First, clinical comparisons were confined to asthma patients without healthy controls, and questionnaire data—often collected during follow-ups—may not fully reflect initial disease status. Therefore, the clinical analysis addressed asthma severity rather than asthma occurrence itself, and objective sleep assessments were not available in this cohort. In addition, the Mendelian randomization analysis was based on chronotype rather than direct circadian disruption. Second, sleep and asthma have a bidirectional relationship: disrupted sleep can worsen asthma, while severe asthma can disturb sleep. Thus, changes in TIM expression may reflect both circadian dysregulation and asthma-related sleep disruption, complicating causal inference. While our murine model revealed a role for TIMin airway remodeling, it did not directly simulate nighttime light exposure, limiting causal inference regarding ALAN and asthma. Lastly, although TIM appears to mediate epithelial and immune responses, its downstream mechanisms remain unclear. These limitations highlight the need for light-controlled models and deeper mechanistic studies to substantiate the circadian regulation of asthma.

## Conclusion

Circadian rhythm disruption due to nighttime light pollution may contribute to the onset and progression of childhood asthma by promoting airway epithelial remodeling. Our multi-level evidence-from population epidemiology to molecular and cellular experiments-suggests that core clock genes such as ***TIM*** may be involved in linking environmental light disturbances on airway epithelial structure. Unlike traditional asthma risk factors such as air pollution or allergens, nighttime light exposure may represent a modern and often overlooked environmental factor that may affect respiratory health in children.

## Data Availability

The raw data supporting the conclusions of this article will be made available by the authors, without undue reservation.

## References

[1] Soriano JB Abajobir AA Abate KH Abera SF Agrawal A Ahmed MB Global, regional, and national deaths, prevalence, disability-adjusted life years, and years lived with disability for chronic obstructive pulmonary disease and asthma, 1990–2015: a systematic analysis for the global burden of disease study 2015. Lancet Respir Med. (2017) 5(9):691–706. 10.1016/S2213-2600(17)30293-X 28822787 PMC5573769

[2] Sun BZ Gaffin JM. Recent insights into the environmental determinants of childhood asthma. Curr Allergy Asthma Rep. (2024) 24(5):253–60. 10.1007/s11882-024-01140-2 38498229 PMC11921288

[3] Agustí A Melén E DeMeo DL Breyer-Kohansal R Faner R. Pathogenesis of chronic obstructive pulmonary disease: understanding the contributions of gene–environment interactions across the lifespan. Lancet Respir Med. (2022) 10(5):512–24. 10.1016/S2213-2600(21)00555-5 35427533 PMC11428195

[4] Kim M Subramanian M Cho Y-H Kim G-H Lee E Park J-J. Short-term exposure to dim light at night disrupts rhythmic behaviors and drivess neurodegeneration in fly models of tauopathy and Alzheimer’s disease. Biochem Biophys Res Commun. (2018) 495(2):1722–9. 10.1016/j.bbrc.2017.12.021 29217196

[5] Falchi F Cinzano P Duriscoe D Kyba CCM Elvidge CD Baugh K The new world atlas of artificial night sky brightness. Sci Adv. (2016) 2(6):e1600377. 10.1126/sciadv.1600377 27386582 PMC4928945

[6] Falchi F Bará S. Light pollution is skyrocketing. Science. (2023) 379(6629):234–5. 10.1126/science.adf4952 36656943

[7] Wang X-L Li L. Circadian clock regulates inflammation and the development of neurodegeneration. Front Cell Infect Microbiol. (2021) 11:696554. 10.3389/fcimb.2021.696554 34595127 PMC8476957

[8] Naik A Forrest KM Paul O Issah Y Valekunja UK Tang SY Circadian regulation of lung repair and regeneration. JCI Insight. (2024) 9(5):e179745. 10.1172/jci.insight.179745 PMC10972589 38456509

[9] Naik A Forrest KM Paul O Issah Y Valekunja UK Tang SY Circadian regulation of lung repair and regeneration. JCI Insight. (2023) 8(16):e164720. 10.1172/jci.insight.164720 37463053 PMC10543710

[10] Myers MP Wager-Smith K Wesley CS Young MW Sehgal A. Positional cloning and sequence analysis of the Drosophila clock gene, timeless. Science. (1995) 270(5237):805–8. 10.1126/science.270.5237.805 7481771

[11] Benna C Scannapieco P Piccin A Sandrelli F Zordan M Rosato E A second timeless gene in Drosophila shares greater sequence similarity with mammalian tim. Curr Biol. (2000) 10(14):R512–3. 10.1016/s0960-9822(00)00594-7 10899011

[12] Cai YD Chiu JC. Timeless in animal circadian clocks and beyond. FEBS J. (2022) 289(21):6559–75. 10.1111/febs.16253 34699674 PMC9038958

[13] Feng K-N Meng P Zou X-L Zhang M Li H-k Yang H-l IL-37 protects against airway remodeling by reversing bronchial epithelial-mesenchymal transition via IL-24 signaling pathway in chronic asthma. Respir Res. (2022) 23(1):244. 10.1186/s12931-022-02167-7 36100847 PMC9472332

[14] Ferrari AJ Santomauro DF Aali A Abate YH Abbafati C Abbastabar H Global incidence, prevalence, years lived with disability (YLDs), disability-adjusted life-years (DALYs), and healthy life expectancy (HALE) for 371 diseases and injuries in 204 countries and territories and 811 subnational locations, 1990–2021: a systematic analysis for the global burden of disease study 2021. Lancet. (2024) 403(10440):2133–61. 10.1016/S0140-6736(24)00757-8 38642570 PMC11122111

[15] Zhang L Ren Z Chen B Gong P Xu B Fu H. A prolonged artificial nighttime-light dataset of China (1984–2020). Sci Data. (2024) 11(1):414. 10.1038/s41597-024-03223-1 38649344 PMC11035565

[16] Truong A Walters A Goodsitt J Hines K Bruss CB Farivar R. Towards automated machine learning: evaluation and comparison of AutoML approaches and tools. 2019 IEEE 31st International Conference on Tools with Artificial Intelligence (ICTAI) (2019). p. 1471–9

[17] Burgess S Dudbridge F Thompson SG. Combining information on multiple instrumental variables in Mendelian randomization: comparison of allele score and summarized data methods. Stat Med. (2016) 35(11):1880–906. 10.1002/sim.6835 26661904 PMC4832315

[18] Liu Y Guo S Sun Y Zhang C Gan J Ning S CRS: a circadian rhythm score model for predicting prognosis and treatment response in cancer patients. J Transl Med. (2023) 21(1):185. 10.1186/s12967-023-04013-w 36895015 PMC9996877

[19] Lane JM Qian J Mignot E Redline S Scheer FAJL Saxena R. Genetics of circadian rhythms and sleep in human health and disease. Nat Rev Genet. (2023) 24(1):4–20. 10.1038/s41576-022-00519-z 36028773 PMC10947799

[20] Pijnenburg MW Fleming L. Advances in understanding and reducing the burden of severe asthma in children. Lancet Respir Med. (2020) 8(10):1032–44. 10.1016/S2213-2600(20)30399-4 32910897

[21] De Marco R Locatelli F Cerveri I Bugiani M Marinoni A Giammanco G. Incidence and remission of asthma: a retrospective study on the natural history of asthma in Italy. J Allergy Clin Immunol. (2002) 110(2):228–35. 10.1067/mai.2002.125600 12170262

[22] Rönmark E Lindberg A Watson L Lundbäck B. Outcome and severity of adult onset asthma–report from the obstructive lung disease in northern Sweden studies (OLIN). Respir Med. (2007) 101(11):2370–7. 10.1016/j.rmed.2007.06.011 17689949

[23] Agustí A Melén E DeMeo DL Breyer-Kohansal R Faner R. Pathogenesis of chronic obstructive pulmonary disease: understanding the contributions of gene-environment interactions across the lifespan. Lancet Respir Med. (2022) 10(5):512–24. 10.1016/S2213-2600(21)00555-5 35427533 PMC11428195

[24] Porsbjerg C Melén E Lehtimäki L Shaw D. Asthma. Lancet. (2023) 401(10379):858–73. 10.1016/S0140-6736(22)02125-0 36682372

[25] Liu J Cao Y Fan T Zhao J Zhu T Gao H The association between outdoor artificial light at night exposure and antenatal depression and anxiety symptoms: a retrospective cohort study in China. Environ Res. (2025) 266:120515. 10.1016/j.envres.2024.120515 39631650

[26] Li J Xu Y Cui W Ji M Su B Wu Y Investigation of nighttime light pollution in Nanjing, China by mapping illuminance from field observations and luojia 1-01 imagery. Sustainability. (2020) 12(2):681. 10.3390/su12020681

[27] Deprato A Maidstone R Cros AP Adan A Haldar P Harding BN Influence of light at night on allergic diseases: a systematic review and meta-analysis. BMC Med. (2024) 22(1):67. 10.1186/s12916-024-03291-5 38355588 PMC10865638

[28] Anenberg SC Henze DK Tinney V Kinney PL Raich W Fann N Estimates of the global burden of ambient, ozone, and on asthma incidence and emergency room visits. Environ Health Perspect. (2018) 126(10):107004. 10.1289/EHP3766 30392403 PMC6371661

[29] Fishbein AB Knutson KL Zee PC. Circadian disruption and human health. J Clin Invest. (2021) 131(19):e148286. 10.1172/JCI148286 34596053 PMC8483747

[30] Giri A Wang Q Rahman I Sundar IK. Circadian molecular clock disruption in chronic pulmonary diseases. Trends Mol Med. (2022) 28(6):513–27. 10.1016/j.molmed.2022.04.002 35508604 PMC9167664

[31] Chen HC Chen YC Wang TN Fang WF Chang YC Chen YM Disrupted expression of circadian clock genes in patients with bronchial asthma. J Asthma Allergy. (2021) 14:371–80. 10.2147/JAA.S302508 33888995 PMC8057829

[32] Wang Y Dong X Pan C Zhu C Qi H Wang Y Single-cell transcriptomic characterization reveals the landscape of airway remodeling and inflammation in a cynomolgus monkey model of asthma. Front Immunol. (2022) 13:1040442. 10.3389/fimmu.2022.1040442 36439114 PMC9685410

[33] Neilsen BK Frodyma DE McCall JL Fisher KW Lewis RE. ERK-mediated TIMELESS expression suppresses G2/M arrest in colon cancer cells. PLoS One. (2019) 14(1):e0209224. 10.1371/journal.pone.0209224 30629587 PMC6328106

[34] Guo SN Jiang XQ Chen N Song SM Fang Y Xie QM Melatonin regulates circadian clock proteins expression in allergic airway inflammation. Heliyon. (2024) 10(6):e27471. 10.1016/j.heliyon.2024.e27471 38496876 PMC10944242

[35] Yin H Wang Z Wang D Nuer M Han M Ren P TIMELESS Promotes the proliferation and migration of lung adenocarcinoma cells by activating EGFR through AMPK and SPHK1 regulation. Eur J Pharmacol. (2023) 955:175883. 10.1016/j.ejphar.2023.175883 37433364

